# Estimating the transmissibility of the 2025 chikungunya fever outbreak in Foshan, China: a modelling study

**DOI:** 10.1186/s40249-025-01376-8

**Published:** 2025-10-16

**Authors:** Yunkang Zhao, Jiahui Li, Zeyu Zhao, Zhiqi Zeng, Beidou Zhang, Yulun Xie, Yanhua Su, Jia Rui, Zifeng Yang, Jianping Huang, Tianmu Chen

**Affiliations:** 1 State Key Laboratory of Vaccines for Infectious Diseases, Xiang An Biomedicine Laboratory, State Key Laboratory of Molecular Vaccinology and Molecular Diagnostics, National Innovation Platform for Industry-Education Integration in Vaccine Research, School of Public Health, Xiamen University, 4221-117 South Xiang’an Road, Xiang’an District, Xiamen City, Fujian Province 361102 People’s Republic of China; 2 KingMed School of Laboratory Medicine, Guangzhou Medical University, Guangzhou City, Guangdong Province 510000 People’s Republic of China; 3 Collaborative Innovation Center for West Ecological Safety, College of Atmospheric Sciences, Lanzhou University, Lanzhou City, Gansu Province 730000 People’s Republic of China; 4 Department of Epidemiology and Health Statistics, Xiangya School of Public Health, Central South University, Changsha City, Hunan Province 410083 People’s Republic of China; 5 State Key Laboratory of Respiratory Disease, National Clinical Research Center Respiratory Disease, Guangzhou Institute of Respiratory Health, The First Affiliated Hospital of Guangzhou Medical University, Guangzhou City, Guangdong Province 510000 People’s Republic of China; 6 Guangzhou Laboratory, Guangzhou City, Guangdong Province 510000 People’s Republic of China

**Keywords:** Vector-borne infections, Modelling, Outbreak, Chikungunya fever, Chikungunya virus, China

## Abstract

**Background:**

Following the onset of an index chikungunya case on July 8, 2025, a significant outbreak occurred in Foshan, Guangdong Province, China. This study aimed to quantify the outbreak's transmissibility between June 16 and July 21, 2025.

**Methods:**

Data were obtained from local Government, Statistics Bureau, Centers for Disease Control and Prevention, and the relevant literature. We employed a transmission dynamic model that integrated human host-vector transmission to estimate the basic reproduction number (${R}_{0}$). The key parameters of the model were calibrated using early-phase limited surveillance data on the cumulative number of cases. We calculated the correlation coefficient to evaluate the accuracy of this calibration. Sensitivity analyses were conducted to quantify the uncertainties in the parameter inputs.

**Results:**

Between June 16 and July 31, 2025, cumulative cases reached 2658, with 92.96% concentrated in the Shunde District. Model simulations showed that a cumulative case count is consistent with local reports (*Pearson r* = 0.99*, P* < 0.001). The median overall ${R}_{0}$ of this outbreak was 7.2807 [interquntile range (IQR): 7.2809‒7.2811], suggesting sustained transmission. Human-to-mosquito transmission (Median: 22.79, IQR: 5.44‒40.14) had a higher median ${R}_{0}$ than mosquito-to-human transmission (Median: 2.33, IQR:0.58‒4.07) (Mann–Whitney U *P* < 0.001). Symptomatic infections (Median: 19.60, IQR: 4.68‒34.52) had a higher median ${R}_{0}$ than asymptomatic infections (Median: 3.19, IQR: 0.76‒5.62) (Mann–Whitney U *P* < 0.001). The model simulated cumulative cases were sensitive to parameters $a$, $b$, ${\omega }_{m}$, and ${\omega }_{p}$. The overall ${R}_{0}$ and mosquito-to-human ${R}_{0}$ were sensitive to parameters $a$ and $b$. The human-to-mosquito and symptomatic human-to-mosquito ${R}_{0}$ were sensitive to parameter $\gamma$, while asymptomatic human-to-mosquito ${R}_{0}$ was sensitive to parameter ${{\omega }_{p}}{\prime}.$

**Conclusions:**

The transmissibility of CHIKV is high. Human-to-mosquito transmission, especially symptomatic infections to mosquito transmission, was the main driver of chikungunya virus transmission. These findings underscore the critical need for enhanced screening of travellers from endemic regions, timely case isolation, and targeted vector control to mitigate autochthonous transmission.

**Graphical abstract:**

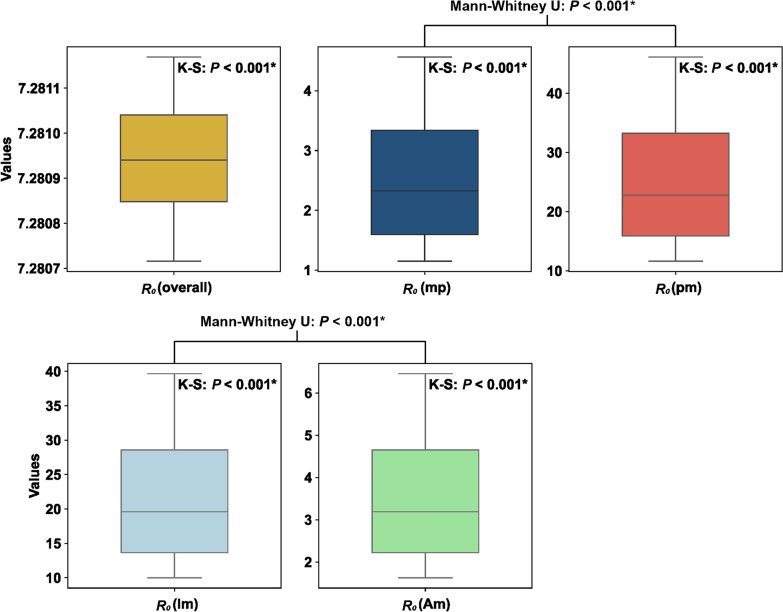

**Supplementary Information:**

The online version contains supplementary material available at 10.1186/s40249-025-01376-8.

## Background

Chikungunya fever is a mosquito-borne disease caused by the chikungunya virus (CHIKV) and is primarily spread by *Aedes* mosquitoes [1, 2]. The virus was first identified in the United Republic of Tanzania in 1952, after which it was sporadically isolated and occasionally caused outbreaks in Africa and Asia [1]. Since 2004, the epidemiology of CHIKV transmission has undergone significant changes, characterised by rapid geographic expansion of the virus [1, 2]. As of December 2024, 119 countries and territories have documented evidence of autochthonous mosquito-borne transmission of CHIKV, which presents an increasing public health challenge [3].

In China, CHIKV was first isolated from the serum of a patient in Yunnan in 1987 [4]. Chikungunya fever is mainly sporadically imported, and small CHIKV outbreaks have been reported, specifically in Guangdong, Zhejiang, and Yunnan Provinces, where *Aedes albopictus*, but not *Aedes aegypti*, were present [3, 4]. However, chikungunya fever is not a notifiable infectious disease; accurate and timely data are limited due to a lack of routine surveillance. Therefore, imported cases are often not detected and managed on time, subsequently leading to the autochthonous transmission [5–8].

Guangdong Province was the first province in China to record autochthonous transmission of chikungunya fever and continues to have the highest number of reported cases [8]. The initial outbreak began in September 2010, lasted for 40 days, and reported 253 infections [8]. The most recent and largest outbreak began on 16 June 2025 following the onset of illness of the index case in Foshan City, and a cluster of cases was reported on July 9. Through rigorous case surveillance and testing, a total of 4824 cases were reported by 26 July 2025 [9, 10].

This study aimed to estimate the transmissibility of the ongoing chikungunya fever outbreak in Foshan City, Guangdong Province, China by utilising limited epidemiological data and applying a mathematical model previously used to analyse the first outbreak in the province [11].

## Method

### Ethics statement

The data for this study were obtained from public databases, news reports, and the relevant literature. The findings were obtained through simulations using mathematical modelling. The study was exempt from ethical review, and the Medical Ethics Committee of Xiamen University waived the requirement for informed consent because (1) all analysed data were anonymised, (2) no medical interventions or biological samples were involved, and (3) the study procedures and results did not affect patient clinical management.

### Study setting and data collection

Foshan City (22°38′N ~ 23°34′N, 112°23′E ~ 113°24′E), a coastal city in Guangdong Province, China, spans 3797.72 square kilometers. Situated near Guangzhou, Shenzhen, Hong Kong, and Macau, it consists of five districts: Chancheng, Nanhai, Shunde, Gaoming, and Sanshui. The subtropical monsoon climate of the city is conducive to *Aedes albopictus* populations. By the end of 2024, the urbanization rate was 95.64% [12].

Demographic data, including population size and birth and death rates, were obtained from the Foshan Municipal Bureau of Statistics’ Statistical Yearbook (Accessed at: https://www.foshan.gov.cn/gzjg/stjj/tjnj_1110962/). Epidemiological information was gathered from a press conference held by the Foshan Municipal People's Government on 22 July 2025 concerning chikungunya fever prevention (Accessed at: https://www.foshan.gov.cn/gzjg/stjj/tjnj_1110962/), which detailed the first reported case, cumulative cases, and spatial distribution as of July 21. Data on mosquito density were collected from the *Aedes albopictus* surveillance report released by the Foshan Center for Disease Control and Prevention (Accessed at: https://yqfk.wjw.gz.gov.cn/wmjcH5/#/home). These reports offer spatial risk indices derived from larval density (Breteau Index, BI) and adult mosquito density (mosquito ovitrap index, MOI). The natural history parameters of chikungunya fever were derived from the published literature and online resources from Hong Kong's Centre for Health Protection (CHP).

### Model development and parameter estimation

We employed a transmission dynamic model that integrated human host-vector transmission to assess the transmissibility of the recent chikungunya fever outbreak in Foshan City, Guangdong Province. This model was employed in the first chikungunya fever outbreak in Guangdong Province in September 2010 [11].

**Fig. 1:**
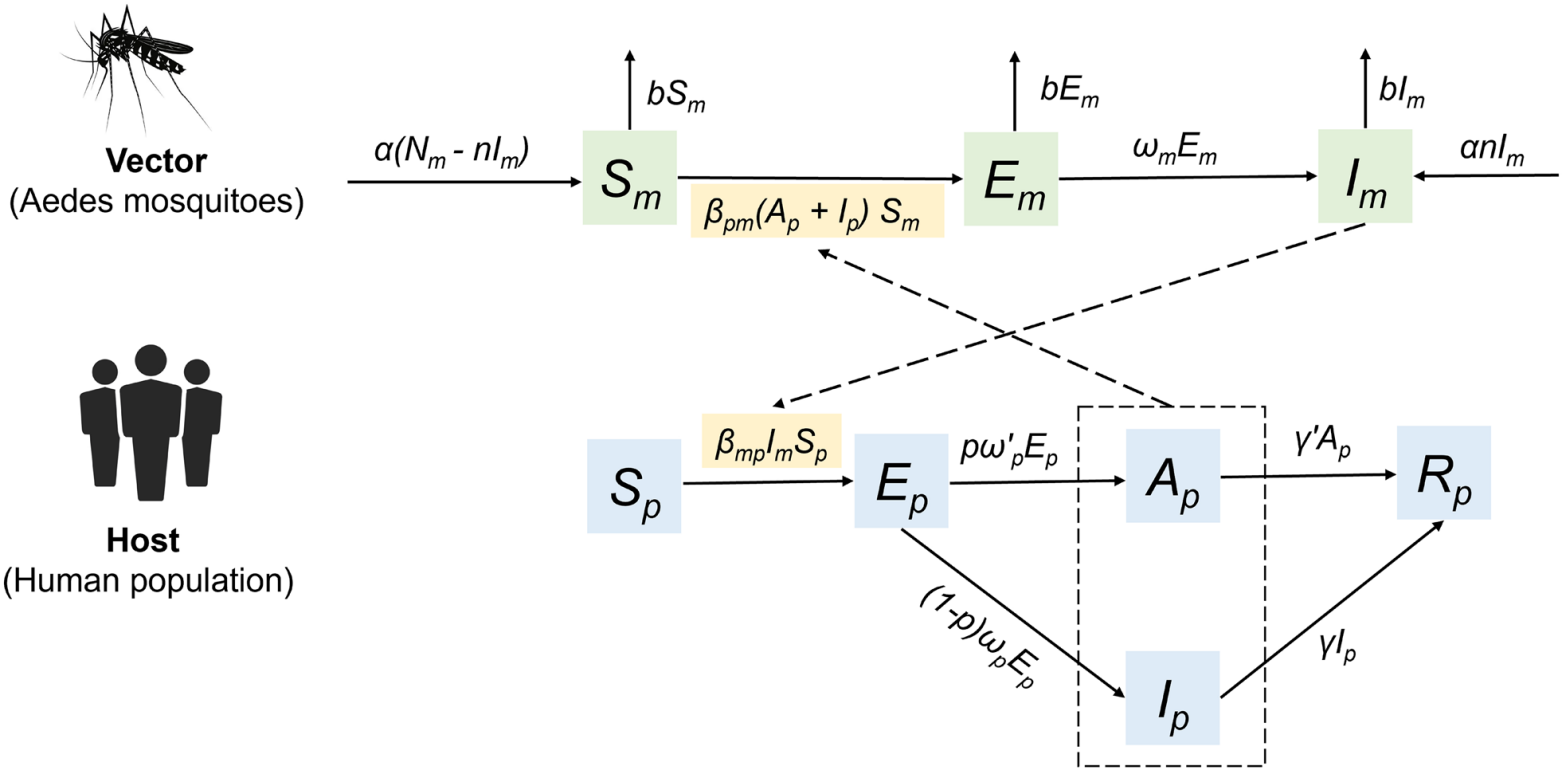
Flowchart of the mathematical model of Chikungunya virus infection

In this model, the human population (${N}_{p}$) was stratified into five compartments: susceptible (${S}_{p}$), exposed (${E}_{p}$), symptomatic infectious (${I}_{p}$), asymptomatic infectious (${A}_{p}$), and recovered (${R}_{p}$). The female *Aedes albopictus* population (${N}_{m}$) was divided into three compartments: (${S}_{m}$), exposed (${E}_{m}$), and infectious (${I}_{m}$) (Fig. 1). The model assumptions were as follows:

(1) Assuming a population with homogeneous susceptibility to the disease, without accounting for reinfections.

(2) Given that Foshan City is an urban environment, our analysis focused exclusively on the human-mosquito transmission cycle, excluding the sylvatic cycle involving non-human primates (NHPs) and mosquitoes.

(3) An imported case initiates transmission via infected mosquitoes biting susceptible humans, generating secondary cases through human-mosquito cycles. Thus, the relative transmission rate from infectious individuals to infectious mosquitoes was ${\beta }_{pm}$, and the relative transmission rate from infectious mosquitoes to infectious individuals was ${\beta }_{mp}$.

(4) After exposure, susceptible individuals progress to either asymptomatic or symptomatic infections following a latent or incubation period. This period is denoted as $${\raise0.7ex\hbox{$1$} \!\mathord{\left/ {\vphantom {1 {\omega_{\rho } }}}\right.\kern-0pt} \!\lower0.7ex\hbox{${\omega_{\rho } }$}}$$ or $${\raise0.7ex\hbox{$1$} \!\mathord{\left/ {\vphantom {1 {\omega_{\rho }^{\prime } }}}\right.\kern-0pt} \!\lower0.7ex\hbox{${\omega_{\rho }^{\prime } }$}}$$, and $$\rho$$ represents the proportion of asymptomatic infections. Once the infectious period concludes, the infectivity of both groups ends, and the virus can no longer be transmitted through mosquito bites. The infectious period of asymptomatic or symptomatic infections was defined as $${\raise0.7ex\hbox{$1$} \!\mathord{\left/ {\vphantom {1 {\gamma^{\prime}}}}\right.\kern-0pt} \!\lower0.7ex\hbox{${\gamma^{\prime}}$}}$$ and $${\raise0.7ex\hbox{$1$} \!\mathord{\left/ {\vphantom {1 \gamma }}\right.\kern-0pt} \!\lower0.7ex\hbox{$\gamma $}}$$.

(5) A certain proportion ($n$) of infected mosquitoes can transmit the virus via vertical transmission. Once *Aedes albopictus* become infected and enter the infectious period, they remain capable of spreading the virus for the rest of their lives.

(6) Assuming that the study population during the outbreak formed a closed and stable cohort without considering births, deaths, and migration.

(7) Transmission through blood transfusions, contact with patient blood, or mother-to-child transmission was not considered, as these occurrences are rare in China according to the Technical Guidelines for the Prevention and Control of Kenyan Fever (2025 Edition).

(8) The birth rate of *Aedes albopictus* in their natural habitat is represented by $a,$ and their death rate by $b$. Assuming that the outbreak's duration is relatively short compared to the disease's long-term persistence, seasonal effects on mosquito population density are disregarded.

(9) When susceptible mosquitoes come into contact with infected individuals, they become infected after an incubation period denoted by $${\raise0.7ex\hbox{$1$} \!\mathord{\left/ {\vphantom {1 {\omega_{m} }}}\right.\kern-0pt} \!\lower0.7ex\hbox{${\omega_{m} }$}}$$.

The model was expressed as follows:

1 $$\begin{array}{c}\frac{d{S}_{p}}{dt}={-\beta }_{mp}{I}_{m}{S}_{p}\end{array}$$

2 $$\begin{array}{c}\frac{d{E}_{p}}{dt}={\beta }_{mp}{I}_{m}{S}_{p}-(1-p{)\omega }_{p}{E}_{p} -p{{\omega }_{p}}{\prime}{E}_{p}\end{array}$$

3 $$\begin{array}{c}\frac{d{I}_{p}}{dt}=(1-p{)\omega }_{p}{E}_{p} - \gamma {I}_{p}\end{array}$$

4 $$\begin{array}{c}\frac{d{A}_{p}}{dt}=p{{\omega }_{p}}{\prime}{E}_{p} - {\gamma }{\prime}{A}_{p}\end{array}$$

5 $$\begin{array}{c}\frac{d{R}_{p}}{dt}={\gamma }{\prime}{A}_{p}+ \gamma {I}_{p}\end{array}$$

6 $$\begin{array}{c}\frac{d{S}_{m}}{dt}=a\left({N}_{m}-n{I}_{m}\right)-{\beta }_{pm}\left({A}_{p}+{I}_{p}\right){S}_{m}-b{S}_{m}\end{array}$$

7 $$\begin{array}{c}\frac{d{E}_{m}}{dt}={\beta }_{pm}\left({A}_{p}+{I}_{p}\right){S}_{m}-{\omega }_{m}{E}_{m}-b{E}_{m}\end{array}$$

8 $$\begin{array}{c}\frac{d{I}_{m}}{dt}={an{I}_{m} + \omega }_{m}{E}_{m}-b{I}_{m}\#\end{array}$$

9 $$\begin{array}{c}{N}_{m}={S}_{m}+{E}_{m}+{I}_{m}\end{array}$$

10 $$\begin{array}{c}{N}_{p}={S}_{p}+{E}_{p}+{I}_{p} +{A}_{p} +{R}_{p}\end{array}$$

Parameter values and plausible ranges were taken from the literature (Table 1). The transmission relative rates ${\beta }_{pm}$, and ${\beta }_{mp}$ were estimated through model simulations to identify combinations that might align with the cumulative infection scale reported for this outbreak. For the incubation and latent period in humans, the infectious periods in humans, and the incubation period in mosquitoes, the median of the range was used, with the corresponding rate determined by taking the reciprocal of the median time as the parameter input for our model. For the proportion of asymptomatic infections in humans and the vertical transmission ratio of mosquitoes, the median of the range was used as the parameter input for our model. The birth and death rates of mosquitoes were directly incorporated into the model using values from Table 1.

**Table 1:** Parameter description and values of the mathematical model

Parameter	Definition	Value	Range	Source
${\beta }_{pm}$	Transmission relative rate from infectious individuals to infectious mosquitoes	–	≥ 0	Model Calibration
${\beta }_{mp}$	Transmission relative rate from infectious mosquitoes to infectious individuals	–	≥ 0	Model Calibration
$${\raise0.7ex\hbox{$1$} \!\mathord{\left/ {\vphantom {1 {\omega_{p}^{\prime } }}}\right.\kern-0pt} \!\lower0.7ex\hbox{${\omega_{p}^{\prime } }$}}$$	Latent period in humans	7 days	2–12 days	[11, 13]
$${\raise0.7ex\hbox{$1$} \!\mathord{\left/ {\vphantom {1 {\omega_{p} }}}\right.\kern-0pt} \!\lower0.7ex\hbox{${\omega_{p} }$}}$$	Incubation period in humans	7 days	2–12 days	[11, 13]
$p$	Proportion of asymptomatic infections in individuals	0.140	0.030–0.250	[9, 11, 17]
$${\raise0.7ex\hbox{$1$} \!\mathord{\left/ {\vphantom {1 \gamma }}\right.\kern-0pt} \!\lower0.7ex\hbox{$\gamma $}}$$	Infectious periods of symptomatic infections in humans	8 days	2–14 days	[11, 13]
$${\raise0.7ex\hbox{$1$} \!\mathord{\left/ {\vphantom {1 {\gamma^{\prime}}}}\right.\kern-0pt} \!\lower0.7ex\hbox{${\gamma^{\prime}}$}}$$	Infectious periods of asymptomatic infections in humans	8 days	2–14 days	[11, 13]
$${\raise0.7ex\hbox{$1$} \!\mathord{\left/ {\vphantom {1 {\omega_{m} }}}\right.\kern-0pt} \!\lower0.7ex\hbox{${\omega_{m} }$}}$$	Incubation period in mosquitoes	6 days	2–10 days	[11–14]
$a$	Birth rate of mosquitoes	0.071	0.000–1.000	[11, 18]
$b$	Death rate of mosquitoes	0.071	0.000–1.000	[11, 18]
$n$	Vertical transmission ratio of mosquitoes	0.168	0.005–0.330	[19]

–: Not applicable

The model's initial parameters and calibration data were informed by the most recent local data available, ensuring the robustness and validity of the model simulations. In the input model, the initial human population size was set to 9.6154 million, as reported in the 2024 Foshan City Statistical Yearbook. The initial number of cases was established as one, occurring on 16 June. The initial number of susceptible individuals was calculated by subtracting the initial number of cases from the total population. All other compartments of humans were set to zero. For *Aedes albopictus*, the initial population was determined using the monthly *Aedes albopictus* surveillance report from the Foshan City Center for Disease Control and Prevention, which provided local average BI and MOI values of 22.94 and 7.06, respectively. We adopted an average value of 15.00 and, following the methodology of previous studies [20, 21], multiplied this value by 20,000 to derive the initial population of the vector. All vectors were considered susceptible in the initial state, meaning that the initial susceptible vector population equalled the initial total vector population, whereas all other compartments were set to zero.

### Simulation method and statistical analysis

We used a calibration-based approach to simulate the combination of transmission relative rates that could replicate the final cumulative cases of this outbreak. To assess the calibration accuracy, we selected an appropriate correlation coefficient (*r*) based on the data distribution.

The basic reproduction number $({R}_{0})$ was used to evaluate the transmissibility of the current chikungunya fever outbreak. We first employed the next-generation matrix methods (NGM) to derive the mathematical expression of ${R}_{0}$ for the model. This expression is further divided to evaluate the transmissibility of human-to-mosquito and mosquito-to-human transmission, as well as that of symptomatic and asymptomatic infections. We calculated the ${R}_{0}$ to evaluate the transmissibility based on transmission relative rates from model calibration. The simulation methods employed, specifically the fourth-order Runge–Kutta method with a tolerance set to 0.001 and a simulated time step of one day, were consistent with those used in previously published studies [22, 23]. The simulation initiation date was determined by subtracting the median incubation period of 7 days from the reported date of the index case on 16 June, resulting in a start date of 9 June [9, 10]. The simulation was conducted from 9 June to 21 July.

${R}_{0}$ was reported as mean (standard deviation, *SD*) or median (interquartile range, IQR), based on the results of normality testing. Statistical tests were chosen appropriately, considering the data type, normality assessment results, and variance homogeneity evaluations. A *P*-value < 0.05 was considered statistically significant [10].

Because most parameters were sourced from the literature and have certain ranges, this introduced uncertainty into the model. Consequently, we conducted a sensitivity analysis to address this issue. This method involved partitioning the literature-derived parameters into 1000 values based on their respective ranges, and then we calculated the mean ± *SD* of the simulated cumulative cases, consistent with previously published studies [22, 23]. Additionally, we further explored the impact of different parameter ranges on ${R}_{0}$ estimates.

Data management was performed using Excel 2019 (Microsoft Corporation, Redmond, USA), whereas data processing and visualisation, model construction, and simulation were conducted in Python 3.9 (Python Software Foundation, Beaverton, USA) via Jupyter Notebook 7.3.2 (Project Jupyter, Austin, USA). The specific Python packages and their versions utilised are detailed in Supplementary Text 1 of the supplementary materials.

## Results

### Derivation of the ${{\varvec{R}}}_{0}$ of the model

Here, the formulated mathematical equations for the ${R}_{0}$ was derived by NGM. We divided ${R}_{0}$ into two components: human-to-mosquito and mosquito-to-human transmissions. Furthermore, human-to-mosquito transmission is categorised into symptomatic and asymptomatic cases. The derivation process of ${R}_{0}$ using the next-generation matrix method [24, 25],the derivation process for ${R}_{0}$ is as follows:

The model defines the total population as${N}_{p}={S}_{p}+{E}_{p}+{I}_{p}+{A}_{p}+{R}_{p}$, the total mosquito population as${N}_{m}={S}_{m}+{E}_{m}+{I}_{m}$. The disease-free equilibrium (DFE) represented by${E}_{0}=\left({N}_{m}, 0 ,0,{ N}_{p}, 0, 0, 0, 0\right)$, in which ${E}_{0m}=\left({N}_{m}, 0, 0\right)$ and${E}_{0p}=\left({N}_{p}, 0, 0, 0, 0\right)$.

Using the next-generation matrix approach, the differential equations for the infectious compartments ${E}_{p}$, ${I}_{p}$, ${A}_{p}$, ${E}_{m}$, and ${I}_{m}$ are decomposed into the inflow matrix $\mathcal{F}$ and the outflow matrix $\mathcal{V}$:

$$\frac{d}{dt}\left[\begin{array}{c}{E}_{p}\\ {I}_{p}\\ {A}_{p}\\ {E}_{m}\\ {I}_{m}\end{array}\right]=\mathcal{F}-\mathcal{V}=\left[\begin{array}{c}{\beta }_{mp}{I}_{m}{S}_{p}\\ 0\\ 0\\ {\beta }_{pm}\left({A}_{p}+{I}_{p}\right){S}_{m}\\ 0\end{array}\right]-\left[\begin{array}{c}(1-p{)\omega }_{p}{E}_{p}+p{{\omega }_{p}}{\prime}{E}_{p}\\ -(1-p{)\omega }_{p}{E}_{p}+ \gamma {I}_{p}\\ -p{{\omega }_{p}}{\prime}{E}_{p}+ {\gamma }{\prime}{A}_{p}\\ {\omega }_{m}{E}_{m}+b{E}_{m}\\ {-an{I}_{m}- \omega }_{m}{E}_{m}+b{I}_{m}\end{array}\right]$$

Taking the partial derivatives with respect to each infectious compartment, we obtain the Jacobian matrices of $\mathcal{F}$ and $\mathcal{V}$ at DFE as follows:

$$F\left({E}_{0}\right)=\left(\begin{array}{ccccc}0& 0& 0& 0& {\beta }_{mp}{N}_{p}\\ 0& 0& 0& 0& 0\\ 0& 0& 0& 0& 0\\ 0& {\beta }_{pm}{N}_{m}& {\beta }_{pm}{N}_{m}& 0& 0\\ 0& 0& 0& 0& 0\end{array}\right)$$

$$V\left({E}_{0}\right)=\left(\begin{array}{ccccc}\left(1-p\right){\omega }_{p}+p{{\omega }_{p}}{\prime}& 0& 0& 0& 0\\ -\left(1-p\right){\omega }_{p}& \gamma & 0& 0& 0\\ -p{{\omega }_{p}}{\prime}& 0& {\gamma }{\prime}& 0& 0\\ 0& 0& 0& {\omega }_{m}+b& 0\\ 0& 0& 0& {-\omega }_{m}& -an+b\end{array}\right)$$

Further solving for the inverse matrix of $V\left({E}_{0}\right)$ yields:

$${V\left({E}_{0}\right)}^{-1}=\left(\begin{array}{ccccc}\frac{1}{\left(1-p\right){\omega }_{p}+p{{\omega }_{p}}{\prime}}& 0& 0& 0& 0\\ \frac{\left(1-p\right){\omega }_{p}}{\gamma \left[\left(1-p\right){\omega }_{p}+p{{\omega }_{p}}{\prime}\right]}& \frac{1}{\gamma }& 0& 0& 0\\ \frac{p{{\omega }_{p}}{\prime}}{{\gamma }{\prime}\left[\left(1-p\right){\omega }_{p}+p{{\omega }_{p}}{\prime}\right]}& 0& \frac{1}{{\gamma }{\prime}}& 0& 0\\ 0& 0& 0& \frac{1}{{\omega }_{m}+b}& 0\\ 0& 0& 0& \frac{{\omega }_{m}}{\left({\omega }_{m}+b\right)\left(-an+b\right)}& \frac{1}{-an+b}\end{array}\right)$$

Then, the basic reproduction number ${R}_{0}$ of model is the spectral radius of the next generation matrix ${FV\left({E}_{0}\right)}^{-1}$. Direct calculation gives:

$$\begin{array}{c}{R}_{0}=\rho \left({FV\left({E}_{0}\right)}^{-1}\right)\\ =\sqrt{\left[\frac{{\beta }_{pm}{N}_{m}\left(1-p\right){\omega }_{p}}{\gamma \left[\left(1-p\right){\omega }_{p}+p{{\omega }_{p}}{\prime}\right]}+\frac{{\beta }_{pm}{N}_{m}p{{\omega }_{p}}{\prime}}{{\gamma }{\prime}\left[\left(1-p\right){\omega }_{p}+p{{\omega }_{p}}{\prime}\right]}\right]}\times \sqrt{\frac{{\beta }_{mp}{N}_{p}{\omega }_{m}}{\left({\omega }_{m}+b\right)\left(-an+b\right)}}\end{array}$$

Furthermore, we break down the ${R}_{0}$ into its components of human-to-mosquito (divided into transmission by symptomatic infectious ${I}_{p}$​, and transmission by asymptomatic infectious ${A}_{p}$​) and mosquito-to-human transmission to assess the transmissibility of the different pathways. The formula is as follows:

- Mosquito-to-human:
  $${R}_{0 (mp)}= \frac{{\beta }_{mp}{N}_{p}{\omega }_{m}}{\left({\omega }_{m}+b\right)\left(-an+b\right)}$$
- Human-to-mosquito:
  $${R}_{0 (pm)}=\frac{{\beta }_{pm}{N}_{m}\left(1-p\right){\omega }_{p}}{\gamma \left[\left(1-p\right){\omega }_{p}+p{{\omega }_{p}}{\prime}\right]}+ \frac{{\beta }_{pm}{N}_{m}p{{\omega }_{p}}{\prime}}{{\gamma }{\prime}\left[\left(1-p\right){\omega }_{p}+p{{\omega }_{p}}{\prime}\right]}={R}_{0 (Im)}+{R}_{0 \left(Am\right)}$$

Among it, ${R}_{0 (Im)}$ represents the part of ${I}_{p}$-to-mosquito, and ${R}_{0 \left(Am\right)}$ represents the part of ${A}_{p}$-to-mosquito.

### Epidemiology, calibration and transmissibility

Since the first imported case was identified on 16 June, Foshan City has reported a cumulative total of 2658 cases as of 21 July. The 92.96% (2471 of 2658) of these cases are concentrated in the Shunde District and no cases were reported in the Gaoming District.

**Fig. 2:**
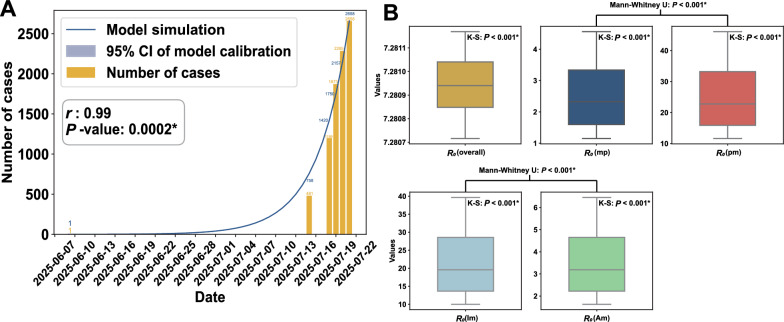
Spatial distribution of cases and model simulation results from June 16 to July 21, 2025. **A** Model simulation of the epidemic curve of the 2025 chikungunya fever outbreak. The blue lines and numbers represent the cumulative number of cases simulated by the model, whereas the yellow bars and numbers show the actual cumulative number of cases. **B** The model estimated the basic reproduction number distribution of the chikungunya fever outbreak. From left to right illustrates the overall basic reproduction number, followed by the basic reproduction numbers for mosquito-to-human, human-to-mosquito, symptomatic human-to-mosquito, and asymptomatic human-to-mosquito transmissions

The model's mean cumulative case count was aligned with the reported cumulative cases (*Pearson r* = 0.99*, P* < 0.001) (Fig. 2A). The model simulates the median ${R}_{0}$ for this outbreak was 7.2807 (IQR: 7.2809‒7.2811). In addition, the median ${R}_{0}$ for human-to-mosquito transmission (Median: 22.79, IQR: 5.44‒40.14) is significantly higher than ${R}_{0}$ for mosquito-to-human transmission (Median: 2.33, IQR:0.58‒4.07) (Levene *P* < 0.001, Mann–Whitney U *P* < 0.001) and for symptomatic human-to-mosquito transmission (Median: 19.60, IQR: 4.68‒34.52) is significantly higher than asymptomatic human-to-mosquito transmission (Median: 3.19, IQR: 0.76‒5.62) (Levene *P* < 0.001, Mann–Whitney U *P* < 0.001) (Fig. 2B).

### Sensitivity analysis for model-simulated cumulative cases and ${{\varvec{R}}}_{0}$

**Fig. 3:**
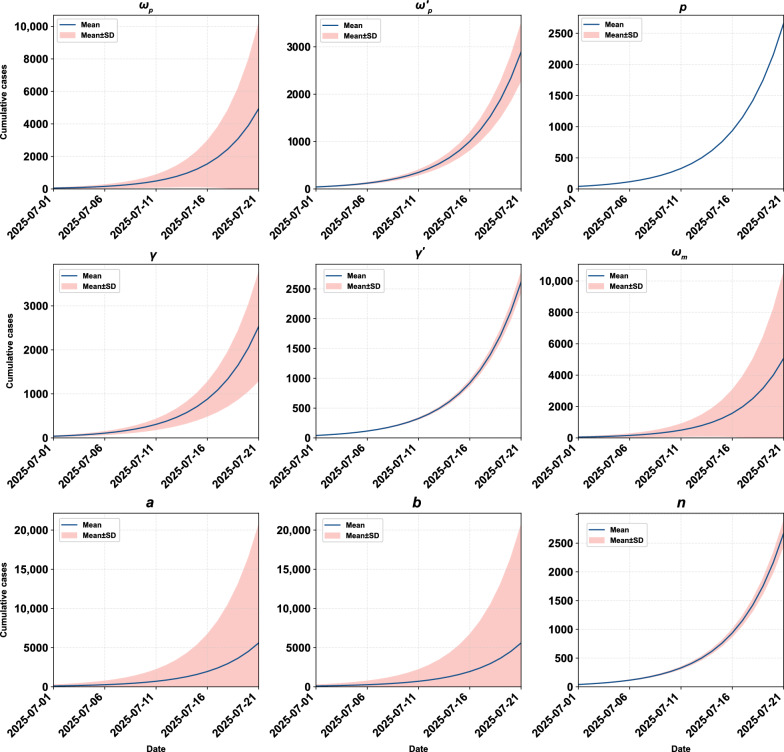
The sensitivity analysis of nine parameters ${\omega }_{p}$, ${{\omega }_{p}}{\prime}$, $p$, $\gamma$, ${\gamma }{\prime}$, ${\omega }_{m}$, $a$, $b$, and $n$ on simulated cumulative number of cases. The red line and the shadow light red band indicate the mean and standard deviation reserved after simulating 1000 times according to the value range of each parameter

The sensitivity analysis found that the model simulated cumulative cases was highly sensitive to parameters $a$, $b$, ${\omega }_{m}$, and ${\omega }_{p}$, moderately sensitive to parameters ${{\omega }_{p}}{\prime}$, $\gamma$, ${\gamma }{\prime}$, and $n$, and not sensitive to parameter $p$ (Fig. 3).

**Fig. 4:**
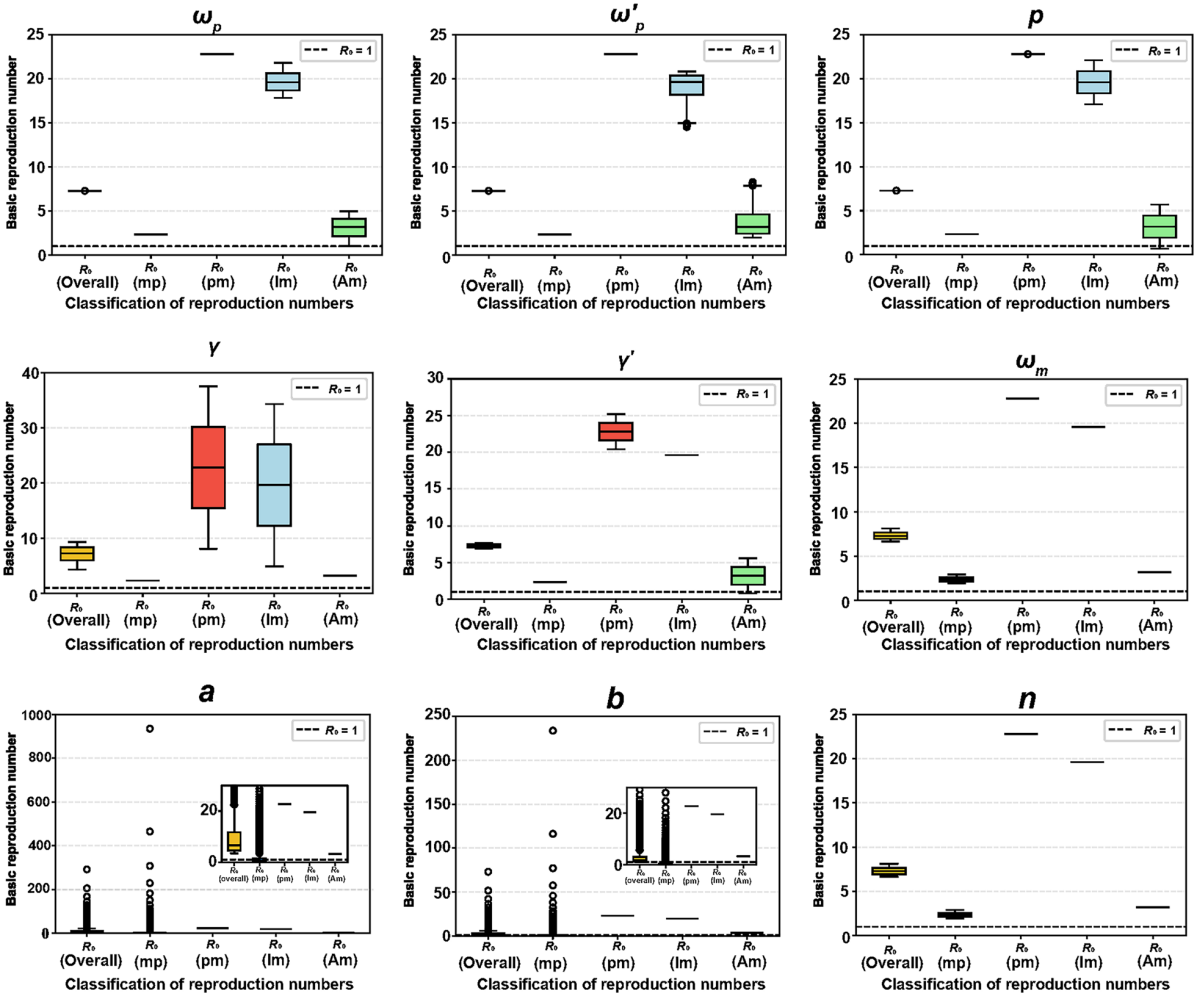
The sensitivity analysis of nine parameters ${\omega }_{p}$, ${{\omega }_{p}}{\prime}$, $p$, $\gamma$, ${\gamma }{\prime}$, ${\omega }_{m}$, $a$, $b$, and $n$ on different basic reproduction numbers. From left to right illustrates the overall basic reproduction number, followed by the basic reproduction numbers for mosquito-to-human, human-to-mosquito, symptomatic human-to-mosquito, and asymptomatic human-to-mosquito transmissions

The sensitivity analysis curves for ${R}_{0}$ were presented in detail in Figure S1. The overall ${R}_{0}$ was sensitive to parameters $a$ and $b$, less sensitive to parameters $\gamma$, ${\gamma }{\prime}$, ${\omega }_{m}$, and $n$, and not sensitive to parameters ${\omega }_{p}$, ${{\omega }_{p}}{\prime}$, and $p$. ${R}_{0}$ for mosquito-to-human transmission was sensitive to parameters $a$ and $b$, less sensitive to parameters ${\omega }_{m}$, and $n$, and not sensitive to other parameters. ${R}_{0}$ for human-to-mosquito transmission was sensitive to parameter $\gamma$, less sensitive to parameter ${\gamma }{\prime}$, and not sensitive to other parameters. ${R}_{0}$ for symptomatic human-to-mosquito transmission was sensitive to parameter $\gamma$, less sensitive to parameters ${\omega }_{p}$, ${{\omega }_{p}}{\prime}$, and $p$, and not sensitive to other parameters. ${R}_{0}$ for asymptomatic human-to-mosquito transmission was sensitive to parameter ${{\omega }_{p}}{\prime}$, less sensitive to parameters ${\omega }_{p}$, $p$, and ${\gamma }{\prime}$, and not sensitive to other parameters (Fig. 4).

## Discussion

This study leverages limited data from the early stages of the outbreak and employs a mathematical model previously used to evaluate the initial outbreak in Guangdong Province [11]. This method allows for a swift estimation of the transmissibility of the 2025 chikungunya fever outbreak in Foshan, Guangdong, China.

We identified Shunde District as the primary epicentre of this outbreak. Our collected cumulative number of casescovering the period from 16 June to 21 July, along with the latest chikungunya fever surveillance information released by the Guangdong Provincial Centre for Disease Control and Prevention, indicates that the number of reported cases in Shunde District significantly exceeded those in other districts [9], supporting our assessment.

We also found that Gaoming District has yet to report any cases. However, considering that other districts in Gaoming District with reported cases experience frequent movement of personnel and materials, and given that this district has no prior reported cases and the entire population lacks immunity, along with a natural meteorological environment similar to other districts conducive to the survival and reproduction of *Aedes albopictus*, Gaoming District also presents a high risk of chikungunya fever transmission. The latest surveillance information reported six new cases in the Gaoming District [9], further supporting our assessment.

The model simulation found a continued rise in the outbreak, with the cumulative number of cases aligned with the official report. This alignment indicates that the simulation calibration results are consistent with the actual reported situation, underscoring the model's reliability in simulating this outbreak.

Our analysis found that the median ${R}_{0}$ for this outbreak was 7.2807 (IQR: 7.2809−7.2811), which is higher than the range of 0.46 6.46 reported in previous studies [26–28]. This variation may be due to several factors, ${R}_{0}$ can vary based on the interaction between the host, vector, and environment for arboviruses as identified in previous studies [26–28]. Most previous studies were conducted in endemic tropical regions where both *Aedes* species are present, whereas our study was conducted in a non-endemic sub-tropical city where only *Aedes albopictus* is present [26–28]. Previous research has shown that the ${R}_{0}$ of mosquito-borne diseases is higher in sub-tropical regions than in tropical regions [26, 27]. Socio-economic disparities across study locations may also affect ${R}_{0}$ estimates [26]. Foshana, a highly developed city in China known for its densely populated urban areas, frequently engages in economic exchanges with Southeast Asia, where chikungunya fever and other mosquito-borne diseases are endemic [29, 30]. As a result, Foshana is one of the most severely affected areas by mosquito-borne diseases in mainland China [30, 31]. Additionally, differences in modelling frameworks, parameter values, and the methodologies for estimation of the ${R}_{0}$ across studies can lead to discrepancies in ${R}_{0}$ estimation. For example, during the 2006 chikungunya fever outbreak on Réunion Island, France, Dumont and Chiroleu estimated ${R}_{0}$ to be between 1.46 and 1.78, while Laith Yakob estimated it to be 4.1 for the same epidemic, likely due to significant differences in the modelling frameworks, parameter values, and estimation methods [32, 33]. Finally, previous studies have estimated ${R}_{0}$ for complete disease outbreak scenarios, whereas the present study estimated ${R}_{0}$ for the early phase of the outbreak, when the number of cases was increasing, which may also contribute to the observed differences in ${R}_{0}$ [26–28].

A recent study employed a generation-time-based maximum likelihood approach for rapid assessment, estimating the ${R}_{0}$ for this outbreak at 16.3, whereas our study estimated it at 7.28 [10]. The ${R}_{0}$ reported in the former study was higher than that estimated in this study and most other regional reports on CHIKV [26–28]. This discrepancy is attributed to the former study's reliance on passive surveillance of legally reported infectious diseases and the assumption of generation time distributions based on published literature [10]. In contrast, this study developed a mechanistic mathematical model based on CHIKV transmission mechanisms that more accurately represents real-world transmission scenarios. However, it is essential to recognise that all models have inherent limitations and estimation uncertainties. Both estimation methods play significant roles in understanding the potential for disease transmission during the early stages. In practical public health decision-making, these approaches are not mutually exclusive; instead, they form a complementary decision support system that collectively provides multidimensional scientific insights for outbreak control. Both estimates indicated an ${R}_{0}$ greater than 1, underscoring the significant transmission dynamic of the outbreak and the urgent need for stringent public health interventions. The integrated data-driven and mechanism-driven methodology addresses the limitations of individual approaches, resulting in more robust ${R}_{0}$ estimates and comprehensive epidemic assessments, thereby facilitating more effective public health decisions.

Our findings revealed that the transmissibility from humans to mosquitoes was significantly higher than that from mosquitoes to humans, and both exceeded the critical threshold. A similar situation occurred during the chikungunya fever outbreak in the summer of 2007 in two neighbouring villages in northeastern Italy [13]. This phenomenon can be attributed to the fact that both this outbreak and the one in France in 2007 resulted from autochthonous transmission following the importation of cases [13]. These imported cases led to the infection of local *Aedes* mosquitoes, which then transmitted the virus to the local population, causing outbreaks. This suggests that in regions where chikungunya fever is not endemic, reducing the risk of human-to-mosquito transmission is a viable strategy for preventing autochthonous transmission events. Measures such as screening mobile populations from endemic regions, early detection and isolation of cases, enhanced mosquito vector surveillance, and control are recommended.

We also found that individuals with symptomatic chikungunya virus infections have a higher transmissibility than those with asymptomatic infections. This may be because Foshan City is not an endemic region for chikungunya fever, making the population generally susceptible and more likely to develop symptoms upon infection, which in turn leads to a relatively low proportion of asymptomatic cases [34, 35]. Nonetheless, the ${R}_{0}$ for both the symptomatic and asymptomatic groups exceeded the critical threshold. Currently, the management of chikungunya fever in China relies on epidemiological investigations and interventions initiated after cases are identified through active medical consultations. Asymptomatic individuals lacking symptoms may not seek medical attention, thereby perpetuating the spread of the virus. Given that Foshan City is not an endemic area, cases often originate from imported instances, leading to autochthonous transmissions [35]. This observation suggests that to mitigate the autochthonous transmission of chikungunya fever, laboratory-based health monitoring should be implemented for individuals returning from endemic regions, addressing the limitations of the current passive surveillance system that depends on active medical consultations.

Sensitivity analysis for simulated cumulative cases revealed that the model was sensitive to the birth rate, death rate, incubation rate and slightly sensitive to the vertical transmission ratio of *Aedes albopictus*. This suggests that monitoring and controlling vectors are crucial factors influencing the scales of the outbreak. Public health campaigns focused on eliminating mosquito breeding sites, such as waterlogged areas, and conducting environmental disinfection to reduce vector density are recommended. Additionally, the model showed sensitivity to the incubation rate of humans and slight sensitivity to the latent rate, infectious rate of humans, indicating that early detection and isolation of cases are vital in affecting the scale of the outbreak. The current disease surveillance method, which depends on active medical visits, may be insufficient for the early identification and control of cases. Therefore, screening and 14-day health monitoring of inbound travellers from endemic regions should be considered. Moreover, proactive contact tracing of identified cases is essential.

Sensitivity analysis revealed that ${R}_{0}$ and ${R}_{0}$ for mosquito-to-human transmission was significantly influenced by Aedes albopictus birth and death rates. This underscored the importance of controlling mosquito vector density as a primary strategy to prevent chikungunya transmission, especially mosquito-to-human transmission. The ${R}_{0}$ for human-to-mosquito transmission was affected by human infectious periods, highlighting the need for prompt case detection and effective case management to disrupt the transmission. The ${R}_{0}$ for symptomatic human-to-mosquito transmission was sensitive to infectious periods, whereas the ${R}_{0}$ for asymptomatic transmission was sensitive to the latent period. This underscored that prevention efforts should prioritise the interruption of human-mosquito contact for symptomatic infections. For asymptomatic cases, the focus should be on early identification through epidemiological investigations and community screening to interrupt human-mosquito contact.

stigations and community screening, thereby interrupting human-mosquito contact.

This study has several limitations. First, owing to constraints in the case surveillance data availability, we were unable to obtain precise daily new case numbers, particularly during the initial stages of the outbreak. Instead of estimating the outbreak's transmissibility through curve fitting of epidemic curves, we employed a method calibrated based on the final infection scale reported by official sources to estimate the transmissibility. The transmissibility estimated using this method may differ from that derived through curve fitting. The estimated average human-to-mosquito relative transmission rate, as determined by this method, was 2.17 × 10^−^⁸ (95% confidence interval: 2.10 × 10^−^⁸ to 2.24 × 10^−^⁸), whereas the average mosquito-to-human relative transmission rate was 2.19 × 10^−^⁸ (2.12 × 10^−^⁸ to 2.26 × 10^−^⁸). However, different formulations of the ${R}_{0}$ and their public health implications, resulting in different sensitivities to changes in the parameters. The overall ${R}_{0}$, which includes the human-mosquito cycle, showed relative insensitivity to changes in relative transmission rates. In contrast, other ${R}_{0}$, which consider only specific critical components of transmission, were more sensitive to variations. Consequently, the overall ${R}_{0}$ presented a narrower interval, whereas the intervals for other ${R}_{0}$ were comparatively broader. Further verification can be conducted when detailed surveillance data is available. The parameters of the model were derived from the existing literature rather than empirical local data, which may introduce some uncertainty into our estimations. In particular, the parameters for mosquito birth and death rates have a relatively significant impact on model estimation, necessitating their priority collection in future routine surveillance and outbreak investigations. Additionally, our model assumes homogeneity in susceptibility across all populations and does not account for adverse outcomes, such as severe illness or mortality. Demographic differences exist in the outcomes of infection, severe illness, and mortality among different populations [36, 37]. We lacked human mobility data. Although the index case suggested that the outbreak was triggered by an imported case infecting local *Aedes* mosquitoes, this limitation may affect the accuracy of our model in simulating real-world conditions.

## Conclusions

The chikungunya fever outbreak in Foshan in 2025 was primarily driven by autochthonous transmission stemming from imported cases, with a notable concentration in Shunde District. The transmissibility of CHIKV is high, and human-to-mosquito transmission, especially symptomatic infections to mosquito transmission, has been identified as the primary factor driving the outbreak. Therefore, it is crucial to enhance the screening and management of individuals from endemic regions, implement timely case tracking and isolation measures, and strengthen vector control efforts to effectively mitigate outbreaks.

## Supplementary Information

Supplementary Material 1.

## Data Availability

The data used in the study can be obtained from the websites mentioned in the methods section, and the code of the study can be accessed based on personal communication with the corresponding authors (chentianmu@xmu.edu.cn).

## Abbreviations

CHIKV: Chikungunya virus
BI: Breteau index
MOI: Mosquito ovitrap index
CHP: Centre for Health Protection
NHPs: Non-human primates
*R*_0_: The basic reproduction number
*r*: Correlation coefficient
*SD*: Standard deviation
IQR: Interquartile range
