# Supplementary material for: Estimating the transmissibility of the 2025 chikungunya fever outbreak in Foshan, China: a modelling study
Source: Infect Dis Poverty. 2025 Oct 16;14:106. doi: 10.1186/s40249-025-01376-8 (PMC12529789; doi:10.1186/s40249-025-01376-8)
Supplement: Supplementary file 1 — Supplementary Material 1. [file 40249_2025_1376_MOESM1_ESM.docx]

**Supplementary Material**

**Estimating the transmissibility of the 2025 chikungunya fever outbreak in Foshan, China: a modelling study**

**Supplementary text 1. Packages Used in This Study**

In this study, we opted to use a Python program for constructing the model simulations, rather than Berkeley Madonna 8.3.18 (Department of Molecular and Cellular Biology, University of California, Berkeley, CA, USA, <http://www.berkeleymadonna.com>), which has been a frequent choice in our previous studies. The Python packages used in this study are listed in **eTable 1**.

**eTable 1. Python packages employed in this study**

| **Names** | **Version** | **Application** |
| --- | --- | --- |
| *pandas* | 1.4.4 | Data import, data clean |
| *numpy* | 1.26.4. | Data Clean |
| *matplotlib* | 3.5.2 | Data visualizations |
| *seaborn* | 0.11.2 | Data visualizations |
| *geopandas* | 1.1.1 | Map visualizations |
| *scipy* | 1.14.1 | Statistical analysis;  Ordinary differential equation solving |
| *joblib* | 1.4.2 | Lightweight pipelining with Python functions |
| *sympy* | 1.13.1 | The next generation matrix method deduces the basic reproduction number |


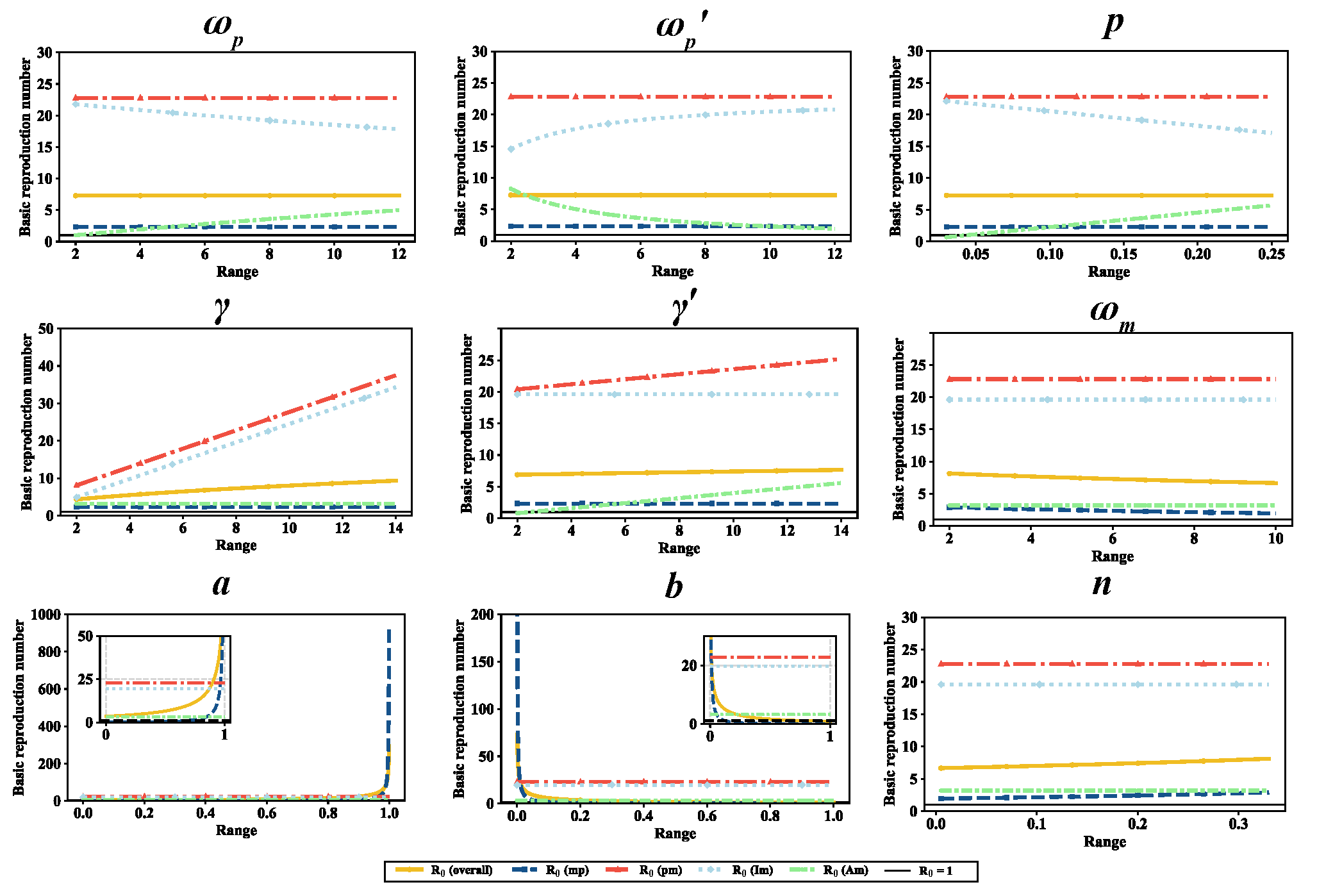


**Figure S1. The sensitivity analysis of nine parameters** $\omega_{p}$**,** ${\omega_{p}}^{'}$**,** $p$**,** $\gamma$**,** $\gamma^{'}$**,** $\omega_{m}$**,** $a$**,** $b$**, and** $n$ **on different basic reproduction numbers.** The solid yellow line represents the overall basic reproduction number ($R_{0}$), the blue dashed line represents the overall $R_{0}$ for mosquito-to-human transmission, the red solid line represents the $R_{0}$ for human-to-mosquito transmission, the light blue dashed lines represent the $R_{0}$ for symptomatic human-to-mosquito transmission, and the green dashed lines represent the $R_{0}$ for asymptomatic human-to-mosquito transmission.
